# Selenomethionine and methyl selenocysteine: multiple-dose pharmacokinetics in selenium-replete men

**DOI:** 10.18632/oncotarget.15460

**Published:** 2017-02-17

**Authors:** James R. Marshall, Raymond F. Burk, Rochelle Payne Ondracek, Kristina E. Hill, Marjorie Perloff, Warren Davis, Roberto Pili, Saby George, Raymond Bergan

**Affiliations:** ^1^ Cancer Prevention and Control, Roswell Park Cancer Institute, Buffalo, NY 14263, USA; ^2^ Division of Gastroenterology, Hepatology, and Nutrition, Department of Medicine, Vanderbilt University Medical Center, C2104 Medical Center North, Nashville, TN 37232, USA; ^3^ National Cancer Institute, Bethesda, MD 20892, USA; ^4^ Department of Medicine, Indiana University School of Medicine, R3 C516, Indianapolis, IN 46202, USA; ^5^ Knight Cancer Institute, Oregon Health Sciences University, Portland, OR 97239, USA

**Keywords:** selenium, selenomethionine, methyl selenocysteine, chemoprevention, pharmacokinetics

## Abstract

According to the Nutritional Prevention of Cancer (NPC) trial, a selenized yeast supplement containing selenium, 200 mcg/day, decreased the incidence of total cancer, cancers of the prostate, colon and lung, and cancer mortality. The active agent in the selenized yeast supplement was assumed to be selenomethionine (SEMET), although the supplement had not been well speciated. The SELECT study, largely motivated by the NPC trial, enrolling nearly 40 times as many subjects, showed unequivocally that selenium 200 mcg/day, with selenium in the form of SEMET, does not protect selenium-replete men against prostate or other major cancer. The agent tested by SELECT, pure SEMET, could have been different from the selenized yeast tested in NPC. One of the selenium forms suspected of having chemopreventive effects, and which may have been present in the NPC agent, is methyl selenocysteine (MSC). This study, with 29 selenium-replete patients enrolled in a randomized, double-blind trial, compared the multiple-dose toxicity, pharmacokinetics and pharmacodynamics of MSC and SEMET. Patients were on trial for 84 days. No toxicity was observed. Although SEMET supplementation increased blood selenium concentration more than MSC did, neither form had a more than minimal impact on the two major selenoproteins: selenoprotein P(SEPP1) and glutathione peroxidase(GPX).

## INTRODUCTION

Selenium (Se) is an essential nutrient [1]; lower Se levels in toenails and blood have been associated in ecological and individual-based epidemiologic studies with increased risk of cancer and a number of other chronic diseases [2–4]. Nonetheless, experimental evidence that selenium supplementation decreases the risk of cancer or of other chronic diseases is mixed at best. The NPC trial, which randomized 1312 patients with a history of nonmelanoma skin cancer to 200 mcg selenium per day in selenized yeast or to a yeast placebo, gave rise to great optimism regarding selenium's chemopreventive properties. NPC patients were treated and followed for an average of 7.4 years, and patients randomized to selenium experienced significantly decreased total cancer incidence, mainly of the lung, colon and prostate; they also experienced significantly decreased total cancer incidence and mortality [5–7]. The association of selenium supplementation with decreased risk was especially marked for prostate cancer [6] and the strongest association of selenium supplementation with decreased risk was noticed among subjects in the lowest tertile of baseline plasma selenium. An important caveat for the NPC findings is that these endpoints were secondary to the primary endpoint: non melanoma skin cancer recurrence. Assignment to supplementation did not decrease, but slightly increased, non melanoma skin cancer recurrence [8].

In a trial testing selenium as a lung cancer prevention agent, Karp and colleagues randomized over 1500 patients with resected non-small cell lung cancer to selenized yeast or to placebo; those patients were chosen to represent a very high risk group. The trial, designed for a 4-year treatment period, was halted after futility analysis showed that the endpoints of second primary tumors and progression-free survival were unlikely to differ in selenium and placebo groups [9] The larger SELECT study, conducted among 35,533 average-risk men, closed early, with subjects followed for an average of 5.5 years. The study was discontinued after the data safety and monitoring committee concluded from futility analysis that there was virtually no possibility that selenium supplementation would be found to decrease the risk of any of the primary endpoints [10]. SWOG 9917, a small study testing whether selenium supplements diminish the progression of high grade prostatic intraepithelial neoplasia to prostate cancer, showed that selenium supplements had no such effect [11].

Comparisons between the NPC, Karp, SELECT and SWOG 9917 studies bring to light two differences of potential importance. First, different forms of Se were used in the trials: SEMET in SELECT and SWOG 9917, selenized yeast in NPC and the Karp trial [5, 8, 9, 11]. A post-trial analysis of the selenium in the analyzed yeast of the NPC showed highly variable amounts of SEMET [12] and several other compounds. A comparison of selenized yeast and SEMET showed that these two agents have approximately equal effects on the generation of two key selenoproteins: selenoprotein P (SEPP1) and glutathione peroxidase (GPX) [13]. A second difference among these trials is that some of the subjects in the NPC trial—especially those in the lowest baseline plasma selenium tertile— were close to being— or were— selenium deficient, while few to none of those in Karp's lung cancer trial, SELECT or SWOG 9917 were [5, 6, 9, 10]. Although the impact of selenium supplementation in NPC appeared to be affected by baseline selenium status, selenium's impact in SELECT and SWOG 9917 appeared not to be [14].

The mechanisms by which selenium might inhibit carcinogenesis or otherwise serve as a chemopreventive agent are not known. It has been hypothesized that a key selenium mechanism involves protection against oxidative stress [15–22]. Those with inadequate selenium stores would, according to this hypothesis, be at increased risk. SEMET is absorbed via methionine transporters and becomes part of the methionine pool [13]. The first steps in methionine metabolism are a methionine cycle and transsulfuration; a fraction of methionine molecules in the mammal are SEMET, so that transsulfuration generates some selenocysteine. Selenocysteine can be converted to selenide, which is critical to synthesis of selenoproteins [23, 24]. Both SEMET and MSC can be transformed to methylselenol, which in cell lines exerts chemopreventive activity [23, 25–27]. Methylselenol, with redox activity and possible effects on signaling in cell lines, has been proposed as a key metabolite in cancer prevention [28], although it may, *in vivo*, simply represent the first step on an excretory pathway [29]. As selenium and sulfur share a number of physiologic characteristics, SEMET can be incorporated into proteins at methionine positions and, thereby, stored as selenomethionine in albumin. Given methionine's importance in proteins, the displacement by selenium of a functionally important sulfur atom has the potential to alter the function of protein-based cellular processes. With continued ingestion, the pool of SEMET accumulates within the body; plasma levels tend to increase until toxicity disturbs metabolism [13]. Approximately 35% of a single 200 mcg dose of Se given as MSC is excreted in urine or feces within 12 days [25]; by contrast, only 15% of the Se in SEMET will have been excreted within that time span [26]. Approximately equal amounts of selenium in MSC are recovered from urine and feces [25], while twice as much selenium given as SEMET is recovered from urine as from feces. Additional amounts are excreted in breath [29]. Unidentified selenium forms may have been present in the NPC yeast [12]. One of these, possibly MSC, may have been at least partly responsible for its apparent chemopreventive effects [30].

Whether any chemopreventive impact of either SEMET or MSC would stem strictly from their impact on these two selenoproteins—or on other selenoproteins—can be argued. Chemopreventive action could result from metabolites of these compounds’ inhibiting of histone deacetylase [31, 32].

A single-dose MSC study over 48 hours showed that Se doses of 400, 800, and 1200 mcg significantly elevated plasma levels of selenium; there was little difference in the dose elevation generated by the 400 and 800 mcg doses [30]. The study did not examine changes in plasma selenoproteins.

The impact of MSC as opposed to SEMET on selenoproteins would help to illuminate the likely physiological importance of these two selenium forms, as well as their possible roles in cancer. It is important, though, that the impact of either agent could result from mechanisms other than the formation of selenoproteins [32–34].

In cell line models, MSC contributes more directly than SEMET to methylselenol [27, 28, 35], which can be demethylated to yield selenide [36, 37] or further methylated to yield dimethyl selenide. Dimethyl selenide is excreted in the breath or, if not released into breath, methylated again to yield trimethyl selenonium and excreted in the urine. It is pertinent to selenium-based chemoprevention to evaluate SEMET and MSC within a single experimental design. The goal of this report was first to test the toxicity of high doses of MSC and SEMET, then to compare and contrast their pharmacokinetics and pharmodynamics in selenium-replete subjects.

## RESULTS

Between 12/20/2011 and 2/13/2014, 29 patients were enrolled in the study. The characteristics of subjects are shown in Table 1. Two extra subjects were accidentally assigned to the 400 mcg dose; their data are included in the results. Variations in the age, pretreatment plasma Se, height, weight and BMI of subjects were within expectations based on chance; none of these demographic or baseline values varied to a statistically significant degree. The ECOG performance status score ranged from 0, meaning that the subject is fully active, to 1, meaning that the subject is restricted in daily activity but able to complete light, sedentary tasks. The mean and median ECOG scores were one, in keeping with the eligibility requirements.

**Table 1:** Participants, phase 1 study of 12-Week treatment by selenomethionine or methylselenocysteine in adult males

		Treatment Group			
Agent	Placebo	Methyl Selenocysteine		Selenomethionine	
Dose		400 mcg	800 mcg	400 mcg	800 mcg
N	3	7	5	5	5
**MEAN ± SD**					
Age (years)	49.3 ± 8	55.6 ± 10	55.9 ± 11	62.2 ± 7	58.9 ± 10
Height (m)	1.8 ± 0.8	1.8 ± 0.1	1.8 ± 0.1	1.8 ± 0.1	1.9 ± 0.0
Weight (kg)	91 ± 20	93 ± 13	90 ± 10	88.9 ± 17	95 ± 10
BMI	28 ± 2	29.2 ± 3	27.7 ± 3	28.6 ± 5	27.4 ± 2.
ECOG Performance Status	Frequency (%)				
'Fully Active'	2 (67)	6 (86)	4 (80)	5 (100)	4 (80)

The primary endpoints were toxicity and changes in biomarkers. In total, 4 adverse events were reported in the 29 eligible subjects who began the study: a grade two hyperbilirubinemia in a placebo participant judged as possibly related to treatment; a grade 2 hyperglycemia in a placebo participant judged as unrelated to treatment ; and two severe adverse events— diagnosis of bladder cancer in an MSC participant, judged unlikely related to treatment; and a stroke diagnosed in an MSC patient, judged unlikely related to treatment. These findings revealed no association between assignment to MSC, SEMET or placebo and the occurrence of adverse or severe adverse events. The 4 patients with adverse or serious adverse events were excluded from the analyses.

Table 2 shows predose values of subjects, by agent and by dose, on days 1, 28 and 84. The overall mean baseline plasma selenium concentration of participants on day 1was 108 mcg/L (not shown). Variability in day 1 predose plasma selenium and SEPP 1 concentrations, and urinary selenium/creatinine ratios was modest enough to be attributed to chance. By days 28 and 84, the Se levels of those receiving 400 and 800 mcg of selenium as SEMET had nearly doubled; the differences from baseline were statistically significant. The day 28 and 84 Se levels of those assigned the 400 mcg dose of MSC had increased by a statistically significant degree; this was not seen in those assigned the 800 mcg dose of MSC. Among those assigned SEMET, values increased appreciably by day 28; by day 84, plasma concentrations had more than doubled for those receiving the 400 mcg dose, while the level among those receiving the 800 mcg dose had nearly tripled. The only statistically significant change in plasma SEPP1 levels observed was on day 28 for subjects assigned to the 800 mcg dose of MSC. By day 84, plasma SEPP1 of these subjects had dropped back, close to baseline.

**Table 2:** Predose selenium and metabolites by agent and by day or study

Treatment Group					
Agent	Placebo	Methyl Selenocysteine		Selenomethionine	
Dose		400 mcg	800 mcg	400 mcg	800 mcg
N	3	7	5	5	5
**Plasma Se concentration (mcg/L)**					
Day 1	106 ± 12	108 ± 15	116 ± 14	105 ± 8	104 ± 14
Day 28	101 ± 7	129 ± 23^1^	128 ± 13	209 ± 36^1^	244 ± 43^1^
Day 84	95 ± 13^1^	123 ± 13^1^	120 ± 12	230 ± 22^1^	291 ± 47^1^
**Plasma Selenoprotein P concentration (mg/L)**					
Day 1	8.0 ± 12	6.9 ± 10	7.5 ± 16	7.0 ± 12	7.5 ± 4
Day 28	7.5 ± 6	7.6 ± 20	8.5 ± 13^1^	6.6 ± 15	7.9 ± 12
Day 84	7.7 ± 17	7.3 ± 12	7.8 ± 12	6.5 ± 12	7.7 ± 10
**Plasma Glutathione Peroxidase activity (Unit/L)**					
Day 1	112 ± 33	112 ± 11	148 ± 34	104 ± 13	132 ± 30
Day 28	115 ± 35	121 ± 16	150 ± 20	125 ± 17	167 ± 52
Day 84	113 ± 45	122 ± 24	185 ± 62	121 ± 16	154 ± 36
**Urine Se /Creatinine ratio (ng Se/mg creatine)**					
Day 1	58.4 ± 55	30.6 ± 10	31.6 ± 9	44.0 ± 12	27.4 ± 11
Day 28	41.3 ± 21	81.4 ± 16	124 ± 29	113 ± 30	142 ± 33
Day 84	61.9 ± 45	90.9^1^ ± 18	124 ± 2^1^	138 ± 39^1^	131 ± 57^1^

^1^Difference from Day 1 *p* <.05 (paired *t* test).

* Mean ± SD.

There was statistically significant variance among the treatment groups in predose, day 1 and day 28 GPX, Nevertheless, although there were changes in GPX activity, none of these was statistically significant. Changes in the ratio of urinary selenium to creatinine take into account a number of extraneous factors, including muscle mass. The pre-dose ratio of urine selenium to creatinine among subjects assigned to MSC or to SEMET, whether 400 or 800 mcg Se, increased significantly by day 84. The differences are noteworthy, with the day 84 levels 3 to 5 times baseline.

Table 3 summarizes the day 1 and day 84 kinetics of plasma Se: maximum concentration (Cmax); time to maximum concentration (Tmax); and area under the curve (AUC). The day 1 Cmax values of each subject did not differ significantly. By day 84, levels of subjects assigned the 400 mcg doses of MSC and SEMET, and those of subjects assigned the 800 mcg dose of SEMET were significantly increased. Variance and changes in the Tmax values of subjects receiving the two species and doses of selenium on days 1 and 84 were within the bounds of what might be expected by chance. The AUC of subjects on day 1 did not vary substantially by Se dose; by day 84, for both the 400 and 800 mcg doses of SEMET, and the 400 mcg dose of MSC, AUC had increased significantly.

**Table 3:** Plasma selenium pharmacokinetics

Treatment Group					
Agent	Placebo	Methyl Selenocysteine		Selenomethionine	
Dose		400 mcg	800 mcg	400 mcg	800 mcg
N	3	7	5	5	5
**Plasma Se Cmax (mcg/L)**					
Day 1	110 ± 9	122 ± 13	131 ± 10	126 ± 12	137 ± 16
Day 84	106 ± 18	136 ± 12^1^	143 ± 18	252 ± 31^1^	319 ± 39^1^
Plasma Se Tmax (hr)					
Day 1	3.67 ± 4	6.43 ± 9	3.50 ± 3	9.20 ± 9	7.40 ± 4
Day 84	2.33 ± 1	3.29 ± 3	2.50 ± 2	3.50 ± 5	2.70 ± 3
**Plasma Se AUC (mcgh/L)**					
Day 1	1170 ± 44	1330 ± 138	1410 ± 132	1370 ± 84	1480 ± 194
Day 84	1160 ± 102	1490 ± 111^1^	1530 ± 183	2780 ± 356^1^	3420 ± 515^1^

^1^Change from day 1 to day 84 *p* < .05 (paired *t* test).

*Mean ± SD.

Table 4 shows SEPP1 kinetics. Cmax, Tmax and AUC for SEPP1 were similar, with no noteworthy variation induced by agent or dose at any point in time. The one statistically significant increase was in Cmax for participants receiving the 400 mcg dose of MSC. It was not seen in the 400 mcg dose of SEMET or the 800 mcg dose of SEMET or MSC. Neither Tmax nor AUC changed significantly by day 84.

**Table 4:** Selenoprotein P pharmacokinetics

Treatment Group					
Agent	Placebo	Methyl Selenocysteine		Selenomethionine	
Dose		400 mcg	800 mcg	400 mcg	800 mcg
N	3	7	5	5	5
**Plasma Selenoprotein P Cmax (mg/L)**					
Day 1	8.37 ± 1	7.84 ± 1	8.62 ± 2	7.56 ± 1	8.38 ± 1
Day 84	8.83 ± 2	8.53 ± 1^1^	8.14 ± 1	7.44 ± 2	8.14 ± 1
**Plasma Selenoprotein P Tmax (hr)**					
Day 1	3.00 ± 4	9.14 ± 7	10.8 ± 8	6.10 ± 10	2.80 ± 3
Day 84	5.00 ± 4	5.07 ± 5	5.80 ± 5	5.00 ± 5	4.90 ± 4
**Plasma Selenoprotein P AUC (mgh/L)**					
Day 1	93.4 ± 11	84.1 ± 9	87.8 ± 16	77.9 ± 12	87.5 ± 11
Day 84	93.9 ± 23	87.1 ± 9	88.7 ± 13	74.3 ± 11	89.6 ± 10

^1^Change from day 1 to day 84 *p* < .05 (paired *t* test).

* Mean ± SD.

Table 5 summarizes pharmacokinetic changes in Gpx activity. None of the changes –in Cmax, Tmax or AUC—was statistically significant.

**Table 5:** Plasma Gpx pharmacokinetics

Treatment Group					
Agent	Placebo	Methyl Selenocysteine		Selenomethionine	
Dose		400 mcg	800 mcg	400 mcg	800 mcg
N	3	7	5	5	5
**Plasma Glutathione Peroxidase Cmax (Units/L)**					
Day 1	202 ± 137	138 ± 23.3	188 ± 49.5	144 ± 30.9	220 ± 116
Day 84	145 ± 71	142 ± 23.3	219 ± 45.7	144 ± 30.2	206 ± 70.2
**Plasma Glutathione Peroxidase Tmax (hr)**					
Day 1	3.33 ± 4.16	4.36 ± 3.77	6.60 ± 10.0	4.00 ± 3.10	6.10 ± 10.0
Day 84	2.00 ± 1.80	2.50 ± 2.16	3.60 ± 4.94	3.20 ± 3.83	6.20 ± 2.86
**Plasma Glutathione Peroxidase AUC (Unitshr/L)**					
Day 1	1560 ± 718	1390 ± 218	1680 ± 372	1370 ± 191	1630 ± 301
Day 84	1430 ± 593	1430 ± 279	1900 ± 166	1430 ± 251	1950 ± 442

* Mean ± SD.

Table 6 provides detail on 12-hour selenium excretion: the total amount of selenium excreted in urine within 12 hours, and the ratio of selenium excreted to the dose assigned. There was no noteworthy or statistically significant change in the amounts of selenium excreted among placebo patients. Among those assigned to selenium, the amounts excreted in urine were substantial. The ratio of selenium excreted within 12 hours to the amount in the dose to which the subject was assigned ranged on day 1 from .15 to .26. By day 84, the ratio was even higher, ranging from .26 to .42.

**Table 6:** Urinary selenium excretion (mcg), day and treatment group

Treatment Group					
	Placebo	Methyl Selenocystene		Selenomethionine	
Day 1		**400 mcg**	**800 mcg**	**400 mcg**	**800 mcg**
Total (0–12 hrs)	27.4 ± 7	67.2 ± 18	147 ± 98	103 ± 38	155 ± 31
Ratio; 0-12 hrsExcretion/dose	N/A	.17	.28	.26	.19
Day 84					
Total (0–12 hrs)	26.0 ± 9	168 ± 45^1^	256 ± 71	156 ± 59^1^	237 ± 54
Ratio; Excretion/dose	N/A	.42	.32	.39	.30

* Mean ± SD.

## DISCUSSION

These data represent the only direct comparison to date of MSC and SEMET as selenium species administered to humans. The goal of this study was to pursue the possibility that MSC might exhibit differential pharmacokinetic and/or pharmacodynamic effects as compared to those of SEMET. A substantial difference, particularly in pharmacodynamics, might have suggested a difference in anticarcinogenic potential. The results indicate that, in selenium-replete subjects, SEMET treatment results in higher blood selenium concentrations than does MSC. Further, the higher day 84 Cmax and AUC values with SEMET, compared to those at day 1, suggest accumulation of selenium with time. Together these findings confirm strong differences in pharmacokinetics of SEMET as opposed to MSC. However, this study failed to identify any meaningful pharmacodynamic differences between SEMET and MSC. Importantly, no pharmacodynamic effects were observed with either SEMET or MSC, when compared to control. It is critical, though, that the measures of pharmacodynamics were limited to changes in two selenoproteins.

Preclinical data indicated that selenium is important in protection against oxidative stress [15–22, 28, 36, 38–40]. Selenium deficiency has also been associated with increased cancer risk [25, 26, 41, 42]. Whether supplementation of those who are selenium deficient decreases vulnerability to oxidative stress is not well known; whether an agent that protects against oxidative stress would protect against carcinogenesis is not known. The Karp trial [9] SELECT [10] and SWOG 9917 [11] document that selenium supplementation by SEMET offers no protection among men who are selenium replete [10].

The NPC study found selenium supplementation to be associated with protection only among subjects in the lowest baseline tertile of plasma selenium [6, 7]. Although an analysis of a subset of SELECT participants found no modification of supplementation effects by baseline selenium status [14], the median baseline serum selenium concentration of those participants was 135 mcg/L, and only 20% of subjects had serum levels below 121.6 mcg/L [10]. (Serum selenium concentrations approximate those in plasma.) In NPC, the mean baseline plasma selenium was 115 mcg/L, and the upper boundary of the lowest tertile was 105 mcg/L. Part of the rationale for the NPC trial was suspected selenium deficiency [9]: selenium status is optimized at a blood level of 85 mcg/L [29]. It is unlikely that more than a small fraction of the participants in SELECT, including those in the lowest percentiles, were at levels in which their selenium status was not optimized. In NPC, some 20 % of subjects had baseline plasma levels below 85 mcg/L (unpublished NPC data). The SEPP1 concentrations and GPX activity levels of subjects in the present trial, whose average baseline plasma selenium concentration was approximately 108 mcg/L—much lower than that of subjects in SELECT, but higher than that of many subjects in NPC—were not affected statistically significantly, or substantially, by supplementation. With plasma in the human optimized at a plasma concentration of approximately 85 mcg/L,selenium above 85 mcg/L is mainly stored in albumin as SEMET [29]. In a subject with a plasma concentration of 108 mcg/L, approximately 85 mcg/L would be selenoproteins, and 23 mcg/L would be SEMET. This study was not able to measure SEMET in plasma. It is possible, however, that, for some trial participants, selenoproteins were below 85 mcg/L. The modest, statistically insignificant increases observed in SEPP1 may indicate that some subjects were marginally deficient in selenoproteins. An effect involving supplication's modification of histone acetylation and deacetylation on carcinogenesis would not necessarily be dependent on baseline selenium levels [31, 32]. Given that the biology of organoselenium compounds is complex and is as yet incompletely understood, other biomarkers that provide more insight are needed.

In light of interest in Se as a possible chemopreventive agent, this study sought as a first goal to assess toxicity. There was no evidence of toxicity. Preclinical toxicology studies of MSC—long and short term— were performed by the National Cancer Institute, through the Division of Cancer Prevention (DCP) Rapid Access to Preventive Intervention Development (RAPID) Program [43, 44]. The studies, conducted in the expectation of chemoprevention based on selenium supplementation, provided little evidence of toxicity at doses well in excess of usual human intake. These findings, providing no evidence of toxicity with treatment by up to 800 mcg/day for 84 days, are consistent with the RAPID results [13, 29].

Selenium is a natural dietary constituent, so that varying baseline concentrations were present in the plasma of subjects. In order to accurately gauge the pharmacokinetic parameters, the analyses took these baseline values into consideration by focusing on changes in means. Supplementation of individuals with plasma selenium above 85 mcg/L will increasingly be excreted, rather than converted into selenoproteins. Although this study did not collect breath or fecal samples, it can be safely assumed that substantial amounts of the selenium supplements ingested were excreted in breath or feces [29].

The findings of this study, in keeping with those of other studies, confirm that selenium supplementation by SEMET leads to selenium accumulation in blood, as selenomethionine is incorporated into proteins at methionine positions; supplementation by MSC does not lead to accumulation in proteins. Selenium supplementation did not substantially affect the generation of the major selenoproteins, SEPP1 or GPX, because the subjects were not selenium deficient [29]. That these subjects were close to being selenium replete is substantiated by the substantial increases in urinary selenium excretion engendered by SEMET and MSC.

The findings are consistent with the passive movement of SEMET into a large storage reservoir. As SEMET accumulates within proteins, with SEMET substituting for methionine, the body provides an unregulated pool. In one previous study, SEMET supplementation for one month by 400 mcg/day of selenium caused the ratio of SEMET to methionine in albumin to increase from 1/8000 to 1/2800 [29]. Nonetheless, intake of selenium in an individual whose selenoproteins are optimized will lead not to the formation of selenoproteins but to selenium excretion; the liver will package selenium as methyl selenol, dimethyl selenide, trimethyl selenonium and selenosugar, and excrete it.

Preclinical, cell-line data indicated that MSC provides a more efficient route than SeMet to the formation of methyl selenol, a metabolite that in cell lines imparts a chemopreventive effect [23, 27, 41, 45]. However, methyl selenol may, *in vivo*, be merely a first step on an excretory pathway [29]. It may be important in the future to accurately speciate methyl selenol and related plasma selenium metabolites *in vivo*; this may be key to the role of selenium in cancer risk. The major downstream protein products of selenium supplementation, SEPP1 and GPX, which are the key and most abundant selenoproteins in plasma, can be readily evaluated. This research has emphasized two selenoproteins to which selenium gives rise [13, 46]. The search for other supplementation biomarkers, however, should not be overlooked.

Ip and others, in research based largely on cell-line analysis, suggested that MSC would be more physiologically relevant to chemoprevention than SEMET or other selenocompounds. It has efficacy in the preclinical models Ip and colleagues studied, and it was therefore hypothesized to represent an important potentially chemopreventive agent [15, 23, 24, 27, 28, 35, 37, 45]. These findings provide little evidence that either form is pertinent to the formation of selenoproteins in individuals who are selenium replete. If either of these agents is active, that action is not seen through the formation of the primary plasma selenoproteins.

The SELECT results leave little room for hope that selenium supplementation, with selenium in the form of SEMET, has chemopreventive efficacy for selenium-replete subjects [10]. Whether supplementation will be of benefit to those who are not selenium replete is less clear; the results reported by the Clark trial suggest that it may be [5–8]. Research on the impact of supplementation to cancer risk will have to be conducted in populations in which selenium deficiency is much more common than in most of the United States. The findings of this study explain little regarding the discrepant findings of NPC, SELECT and SWOG 9917; the most plausible explanation is that, if selenium has preventive value, it must either be for individuals whose selenium status is less than optimal, or by means of MSC or another formulation of selenium.

## MATERIALS AND METHODS

### Participants

An IRB approved, Phase I multiple-dose, dose-escalation pharmacokinetic/toxicity study of SEMET and MSC was conducted at Roswell Park Cancer Institute (RPCI). Healthy male volunteers were recruited as subjects by public announcement in Buffalo, NY. After granting informed consent verbally and in writing, subjects were randomized, double-blinded, to receive daily doses of either MSC or SEMET, either 400 or 800 mcg Se/day, or placebo, for 84 days. In the first wave, 5 patients were assigned to 400 mcg of selenium as MSC, 5 to the same selenium dose as SEMET and two to placebo; in the second wave, 5 patients were assigned to receive 800 mcg of selenium as MSC, five to the same selenium dose as SEMET, and two to placebo. The goal of placebo-group inclusion was to decrease the likelihood of confounding by participant reporting of inconsequential, subjective symptoms of toxicity. The placebo arm is displayed in the results, although the placebo data are not included in statistical analyses. With a total of only 3 placebo patients, and only 5–7 patients in each selenium-supplementation group, precision of comparisons to placebo patients is extremely limited the treatment groups—400 mcg Se as MSC; 400 mcg Se as SEMET; 800 mcg Se as MSC; 800 mcg Se as SEMET—will be referenced as 400 or 800 mcg: these refer to the amount of selenium, rather than to the amount of SEMET or MSC, in the doses.

Subjects were required to have normal hepatic, renal and bone marrow function as assessed by history, physical, and clinical chemistry analysis. They could not have donated blood within 30 days of first blood sampling, had to be 18 or older, to have an Eastern Cooperative Oncology Group (ECOG) performance status [47] of 0 or 1, and to weigh between 50 and 115 kg. Eligibility was restricted to males, because the most convincing association of selenium supplementation with reduced risk in the NPC study was seen with prostate cancer. Subjects could not be taking prescription or nonprescription drugs, vitamins or herbal supplements known to affect gastric acidity within three days of agent administration.

**Figure 1:**
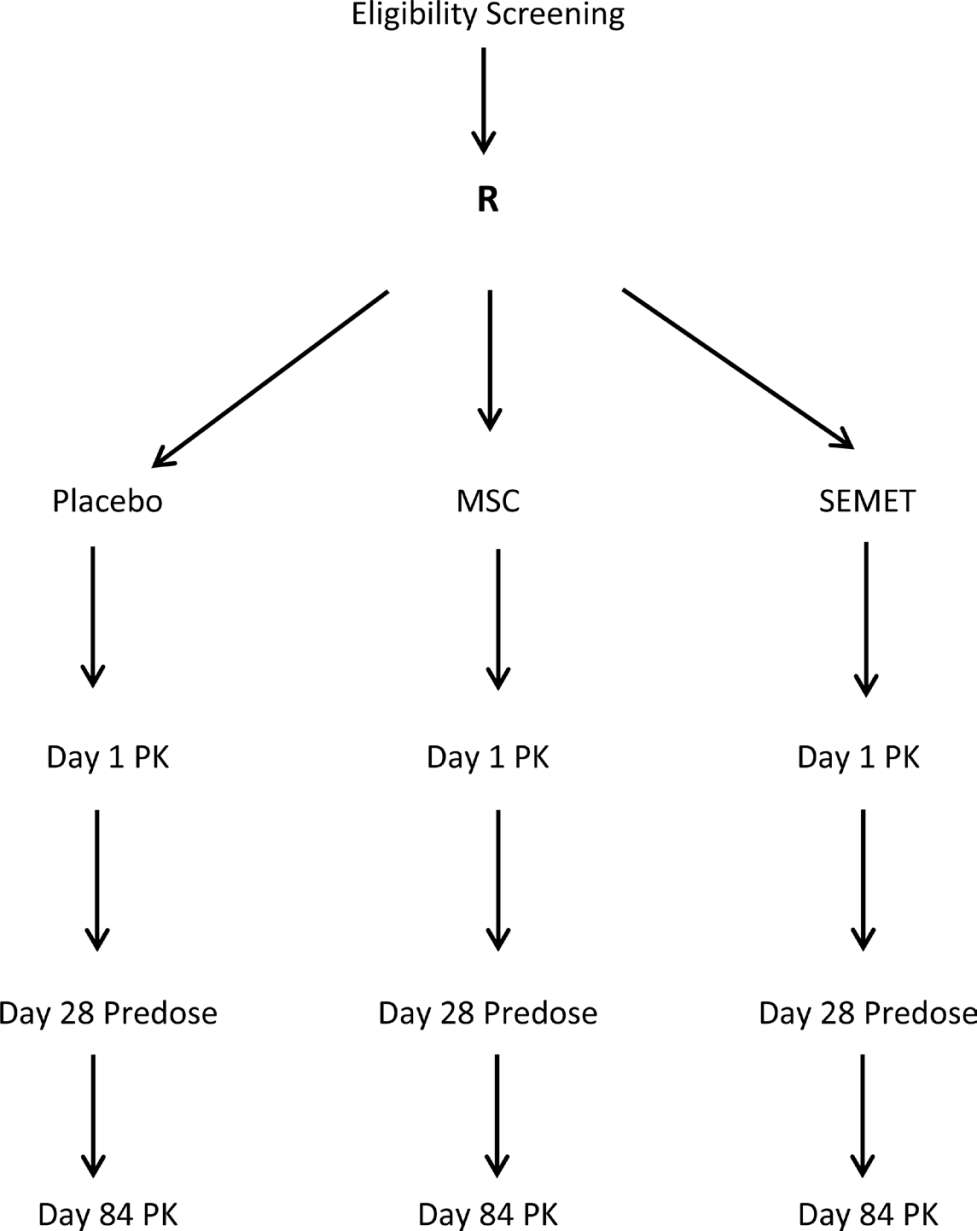
Phase 1 Multiple Dose Study of 12-week treatment by Se=Methyl-L-Selenocysteine (MSC) and L-Selenomethionine (SEMET) in adult males

Pharmacokinetic analysis was undertaken on days 1 and 84. Subjects arrived at RPCI at 7:00 am on days 1 and 84 after a fast that began at 10:00 pm the previous night. After a brief review of concurrent medications and vital signs and symptoms, subjects had an intravenous catheter placed in one arm. A baseline pre-dose blood sample was drawn through the catheter, after which subjects ingested the assigned agent along with 8 ounces of water under direct supervision. Subjects remained in hospital for 12 hours, returning at 24 hours. Blood was drawn previous to dosing at baseline and at 0.5, 1.0, 1.5, 2.0, 3.0, 4.0, 6.0, 8.0, 10.0, 12.0 and 24 hours after the first day's dosing. A predose blood sample was collected on day 28. On day 84, blood was drawn as on day 1 up to 12 hours after dosing. Each sample was treated with 1 mg of disodium EDTA per ml to prevent coagulation. Plasma was obtained by centrifugation. Urine output was collected within time spans of 0–4, 4–8 and 8–12 hours. Figure 1 summarizes this schema. All participants in the 400 mcg groups were treated and evaluated for toxicity prior to treating those in the 800 mcg groups. The occurrence of grade 2 or greater toxicity thought at least possibly due to agent was set to preclude escalation to the next higher dose.

Plasma and urine selenium concentrations and plasma SEPP1 concentration and GPX activity were determined by the methods of Burk et al [13]. Selenium in urine was measured by the ratio of selenium to creatinine, and by urinary excretion of selenium.

### Methods/statistics

Toxicity was evaluated in all subjects. History was reviewed at baseline; physical examinations were conducted at screening, on days 1, 28 and 84. Medications were reviewed at screening, days 1, 2, 3, 28 and 84. Participants were contacted by telephone for weight change and gastrointestinal distress, with all toxicities recorded, on days 14, 45, 60 and 112. Vital signs were checked at screening, day 1, 2, 3, 28, and 84. Clinical laboratory studies, including SGOT/SGPT, total bilirubin, serum electrolytes with BUN, TSH, T4 and creatinine, fasting blood sugar, lipid panel (total cholesterol, HDL, LDL, triglycerides), PSA and urinalysis, were performed at screening, day 1, day 28 and day 84. Participants were contacted by telephone 30 days after their participation was complete for toxicity assessment. Toxicities for all consented subjects were recorded and graded according to the National Cancer Institute Common Toxicity Criteria (CTC) version 3.0 [47].

Quantitative descriptors of subjects—age, baseline plasma selenium, height, weight and ECOG [47] performance status—were compared by means and standard deviations. One-way analysis of variance (ANOVA) was used to identify the statistical significance of variance among the group means. These comparisons do not include the placebo group. Because the focus of this trial was on changes in biomarkers, statistical significance emphasized changes in means, tested by paired *t* tests. As the statistical power of comparisons was limited by the small number of patients in any one group, interpretation of the findings is necessarily conservative. The .05 level of statistical significance was used, and all analyses were two-tailed.

## CONCLUSIONS

This study confirms that, for men with adequate selenium nutriture, as indicated by plasma selenium concentrations in the vicinity of 108 mcg/L, 12 week supplementation by 400 and 800 mcg of selenium, whether by MSC or SEMET, does not lead to toxicity. MSC and SEMET demonstrate differential pharmacokinetics. Neither form, however, has any impact on the formation of the major selenoproteins: SEPP1 and GPX. If selenium supplementation has any chemopreventive activity among selenium replete subjects, it is probably by mechanisms other than the formation of these two selenoproteins.
